# Supplementary material for: Identification of photoperiod-induced specific miRNAs in the adrenal glands of Sunite sheep (Ovis aries)
Source: Front Vet Sci. 2022 Jul 22;9:888207. doi: 10.3389/fvets.2022.888207 (PMC9354845; doi:10.3389/fvets.2022.888207)
Supplement: Supplementary file 1 [file Data_Sheet_1.ZIP › Supplementary file/miRDeep2 file.zip!/miRDeep2 file/Know_Structure/SP42A3/oar-mir-106b.pdf]

miRBase precursor : oar-mir-106b
Total read count : 3515
oar-miR-106b read count : 1325
remaining reads : 2190

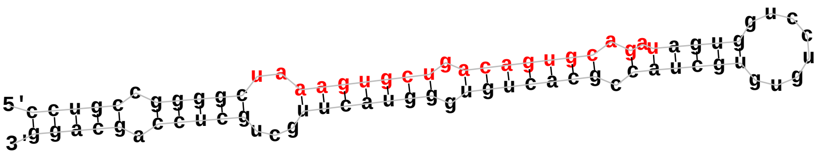

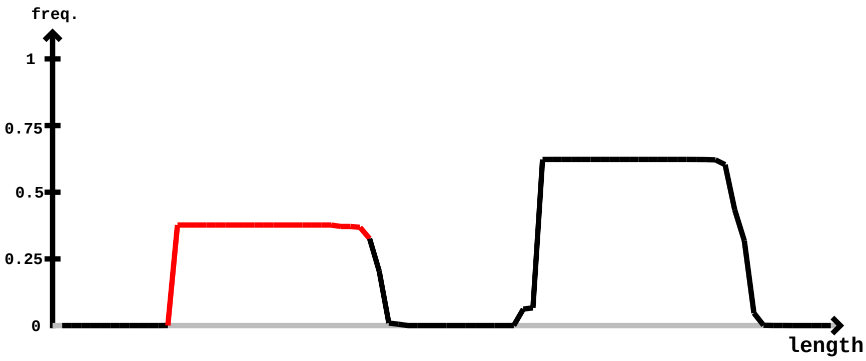

oar-miR-106b

5'- ccugccggggcuaaagugcugacagugcagauaguggucc ugugugcuaccgcacuguggguacuugcugcuccagcagg -3'	exp		
((((.(((((..(((((((.(((((((.(.(((((........)))))).))))))).)))))))...))))).)))))	reads	mm	sample
..........cuaaagugcugacagugcagaua...	1	0	seq
...........uaaagugcugacagugc..	15	0	seq
...........uaaagugcugacagugA..	3	1	seq
...........uaaagugcugacaguUc..	1	1	seq
...........uaaagugcugacagugcag..	11	0	seq
...........uaaagugcugacagugcaga...	149	0	seq
...........uaaagugcugacagugcagau..	429	0	seq
...........uaaagugcugacagugcagaua...	683	0	seq
...........uaaagugcugacagugcagauag..	15	0	seq
...........uaaagugcugacagugcagauagu...	18	0	seq
....uaccgcacuguggguacu...............	1	0	seq
....uaccgcacuguggguacuu..............	1	0	seq
....uaccgcacuguggguacuug.............	1	0	seq
....uaccgcacuguggguacuugc............	13	0	seq
....uaccgcacuguggguacuugcu...........	198	0	seq
....uaccgcacuguggguacuugcug..........	1	0	seq
....uaccgcacuguggguacuugcugc.........	2	0	seq
....uaccgcacuguggguacuugcugcu........	1	0	seq
....accgcacuguggguacuug.............	1	0	seq
....accgcacuguggguacuugcu...........	8	0	seq
....accgcacuguggguacuugcug..........	3	0	seq
....accgcacuguggguacuugcugc.........	3	0	seq
....ccgcacuguggguacuug.............	2	0	seq
....ccgcacuguggguacuugc............	50	0	seq
....ccgcacuguggguacuugcu...........	388	0	seq
....ccgcacuguggguacuugcug..........	404	0	seq
....ccgcacuguggguacuugcugc.........	948	0	seq
....ccgcacuguggguacuugcugcu........	160	0	seq
....ccgcacuguggguacuugcugcuc.......	3	0	seq
....ccgcacuguggguacuugcugcucca.....	1	0	seq
....cgcacuguggguacuugcugc.........	1	0	seq
